# Gene expression of oxidative stress markers and lung function: A CARDIA lung study

**DOI:** 10.1002/mgg3.1832

**Published:** 2021-11-19

**Authors:** Ramya Ramasubramanian, Ravi Kalhan, David R. Jacobs, George R. Washko, Lifang Hou, Myron D. Gross, Weihua Guan, Bharat Thyagarajan

**Affiliations:** ^1^ Division of Epidemiology and Community Health University of Minnesota School of Public Health Minneapolis Minnesota USA; ^2^ Department of Preventive Medicine Northwestern University Feinberg School of Medicine Chicago Illinois USA; ^3^ Division of Pulmonary and Critical Care Medicine Northwestern University Feinberg School of Medicine Chicago Illinois USA; ^4^ Division of Pulmonary and Critical Care Medicine Brigham and Women’s Hospital Boston Massachusetts USA; ^5^ Applied Chest Imaging Laboratory Brigham and Women’s Hospital Boston Massachusetts USA; ^6^ Department of Pathology and Laboratory Medicine University of Minnesota School of Medicine Minneapolis Minnesota USA; ^7^ Department of Biostatistics University of Minnesota School of Public Health Minneapolis Minnesota USA

**Keywords:** CARDIA, gene expression, lung function, oxidative stress

## Abstract

**Background:**

Circulating markers of oxidative stress have been associated with lower lung function. Our objective was to study the association of gene expression levels of oxidative stress pathway genes (*ALOX12*, *ALOX15*, *ARG2*, *GSTT1*, *LPO*, *MPO*, *NDUFB3*, *PLA2G7*, and *SOD3)* and lung function forced expiratory volume in one second (FEV_1_), forced vital capacity (FVC) in Coronary Artery Risk Development in Young Adults study.

**Methods:**

Lung function was measured using spirometry and the Nanostring platform was used to estimate gene expression levels. Linear regression models were used to study association of lung function measured at year 30, 10‐year decline in lung function and gene expression after adjustment for center, smoking, and BMI, measured at year 25.

**Results:**

The 10‐year decline of FEV_1_ was faster in highest *NDUFB3* quartile compared to the lowest (difference = −2.09%; *p* = 0.001) after adjustment for multiple comparisons. The 10‐year decline in FEV_1_ and FVC was nominally slower in highest versus lowest quartile of *PLA2G7* (difference = 1.14%; *p* = 0.02, and difference = 1.06%; *p* = 0.005, respectively). The other genes in the study were not associated with FEV_1_ or FVC.

**Conclusion:**

Higher gene expression levels in oxidative stress pathway genes are associated with faster 10‐year FEV_1_ decline.

## Graphical abstract

Higher levels of oxidative stress biomarkers have been associated with reduced lung function. This study found that among 2527 participants in the CARDIA study higher peripheral blood gene expression levels in oxidative stress pathway genes such as NDUFB3 are associated with faster 10‐year FEV1 decline. Thus, gene expression levels may be a useful biomarker to estimate oxidative stress in population studies.

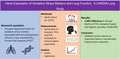

## 1 INTRODUCTION

Oxidative stress, the imbalance between oxidant and antioxidant effects in the body, is associated with asthma and chronic pulmonary obstructive disease (COPD; Ahmad et al., 2012; Hecker, 2018; Holguin, 2013; Montuschi et al., 2000; Ochs‐Balcom et al., 2006; Park et al., 2009). Specifically, pro‐oxidants such as p‐TBARS have been associated with lower lung function (Mannino et al., 2003; Sircar et al., 2007) and antioxidants such as carotenoids were positively associated with forced expiratory volume in one second (FEV_1_) and forced vital capacity (FVC) in cross‐sectional studies (Ochs‐Balcom et al., 2005; Schunemann et al., 1997).

Though oxidative stress is determined by a regulation of complex biological processes, the release of reactive oxygen species (ROS) is an important mechanism for increasing oxidative damage while the activities of various antioxidant enzymes are important defenses against oxidative damage. Increased ROS production can occur through several mechanisms that include the electron transport chain (ETC) in mitochondria, (Droge, 2002; Papaharalambus & Griendling, 2007) increased production of superoxides (e.g.,) ARG2, short‐lived oxidized intermediates such as hypochlorous acid from myeloperoxidase (MPO) and hypothiocyanite from lactoperoxidase (LPO) or from intermediates in lipid metabolism such as lipid peroxidation catalyzed by lipoxygenases such as ALOX12 and ALOX15 or lipid hydrolysis catalyzed by platelet‐activating factor acetohydrolase (PAF‐AH; Gago‐Dominguez et al., 2007; Pierini & Bryan, 2015). In addition to increased ROS production, lower activity of antioxidant defenses such as inadequate antioxidant enzyme concentrations such as glutathione transferases (GSTs) and superoxide dismutases (SODs) that metabolize products derived from oxidative stress such as superoxides, lipids, and DNA products can also result in increased oxidative stress (Kruse et al., 2000; Singh & Bhat, 2012; Suwanpradid et al., 2014; Wang et al., 2018). Thus, measurement of gene expression levels of enzymes involved in both increasing oxidative stress as well as maintaining antioxidant defenses can help us better understand the influence of these pathways on pulmonary function and disorders. Thus, we specifically evaluated expression of candidate genes in major pathways contributing to oxidative stress. We evaluated seven genes that increase ROS production and two genes involved in antioxidant defenses. The seven genes involved in increased ROS production include *NDUFB3*, a subunit of complex I and the largest complex in ETC (Calvo et al., 2012; Haack et al., 2012; Leman et al., 2015), *ALOX12*, and *ALOX15* that are involved in lipid peroxidation (Brash, 1999; Mashima & Okuyama, 2015; Pallast et al., 2009; Praticò et al., 2004; Seiler et al., 2008; Suzuki et al., 2015), *PLA2G7* that is involved in lipid hydrolysis (Miwa et al., 1988; Stafforini, 2009; Stafforini et al., 1999, 2006), and *ARG2* (Suwanpradid et al., 2014; Yang & Ming, 2014), *MPO* and *LPO* (Anatoliotakis et al., 2013; Aratani, 2018; Stamp et al., 2012) that form short‐lived intermediate free radicals.

In this article, we will study the associations between gene expression of the nine oxidative stress markers and pulmonary function defined by FEV_1_ and FVC in the Coronary Artery Risk Development in Young Adults (CARDIA) study. We hypothesized that higher expression levels of genes that increase oxidative stress and lower expression of antioxidant genes would be associated with a lower lung function measurement, and with a faster decline in lung function.

## 2 METHODS

### 2.1 Study population

#### 2.1.1 Ethical compliance

All study methods were carried out in accordance with relevant guidelines and regulations. All CARDIA participants provided a signed informed consent before study participation and sign a new informed consent form at every examination.

CARDIA is a cohort study with 5115 participants who were recruited at baseline examination during the year 1985–1986 at four field centers (Birmingham, AL; Chicago, IL; Minneapolis, MN; and Oakland, CA). The study included approximately equal number of Blacks and Whites; men and women, respectively. The follow‐up rates in CARDIA are 72% at year 20 (2005–2006) and year 25 (2010–2011), and 71% at year 30 (2015–2016). The detailed methods, instruments, and quality control procedures for the CARDIA study have been previously described (Friedman et al., 1988; Hughes et al., 1987). All study methods were carried out in accordance with relevant guidelines and regulations. All CARDIA participants provided a signed informed consent before study participation and sign a new informed consent form at every examination.

The cross‐sectional analyses performed to study associations between year 25 gene expression levels and year 30 lung function measurements included 2527 participants. The longitudinal analyses performed to study associations between 10‐year decline in lung function from year 20 to year 30 and year 25 gene expression levels included 2271 participants. Participants with missing lung function data, missing gene expression measurements, and missing covariates were removed prior to analysis (Ramasubramanian et al., 2020). We performed the sensitivity analysis by removing participants with COPD and asthma when evaluating the association between year 25 gene expression levels and year 30 lung function. For sensitivity analyses, 55 participants with COPD and 476 participants with asthma were removed for the cross‐sectional analysis while 47 participants with COPD and 442 participants with asthma were removed from the longitudinal analysis.

### 2.2 Spirometry

Spirometry was performed using a dry rolling‐seal OMI spirometer (Viasys Corp, Loma Linda, CA) at year 20 examination and a portable spirometer EasyOne Diagnostic, NDD Medical Technologies, Andover, MA) at year 30 following the American Thoracic Society Guidelines (Miller et al., 2005).

### 2.3 Gene expression analysis

Whole blood was collected in the PAXgene Blood RNA tubes (Qiagen Inc.) at the year 25 examination. mRNA was isolated using the PAXgene Blood RNA kit (Qiagen Inc.) at the Molecular Epidemiology and Biomarker Research Laboratory (MEBRL) according to the manufacturer's instructions. The detailed methods for measurement and normalization of gene expression using the nCounter analysis system (Nanostring Inc.) were published previously (Ramasubramanian et al., 2020). Briefly, normalization of the gene expression was done with a combination of positive control normalization, housekeeping gene normalization, and CodeSet content normalization to correct major sources of error including pipetting errors, instrument scan resolution, batch variations, and sample input variability. Specifically, both positive control normalization and the CodeSet content normalization help to adjust for batch variation and assay variation related to specific reagents and beads used in the nCounter analysis system (Nanostring Inc.). The raw counts of the gene expression of sample were first multiplied by the sample‐specific positive control normalization factor, then by the housekeeping gene normalization factor, and the CodeSet normalization factor to obtain the final gene expression counts.

### 2.4 Measurement of covariates

The covariates used for this analysis are smoking and BMI. Smoking was determined using a pack‐years variable which was measured by cigarette pack‐years (cigarette packs smoked per day × number of years smoking). BMI was defined as a continuous variable and was calculated as weight (kg) divided by height (meters) squared. Year 25 measurements of BMI and smoking status were used in this analysis.

### 2.5 Statistical methods

Characteristics of participants at year 25 among five levels of the nine genes were assessed by using Chi‐square tests for categorical variables and one‐way ANOVA for continuous variables. The lower limit of detection for the gene expression counts was set at 16 and all counts lower than the lower limit of detection was set 16 prior to analysis. The gene expression of *ALOX12*, *ALOX15*, *ARG2*, *GSTT1*, *LPO*, *MPO*, *NDUFB3*, *PLA2G7*, and *SOD3* were divided into quartiles. Linear regression models were used to evaluate the association of predicted lung function at exam year 30 and 10‐year decline in lung function (from year 20 to year 30) with year 25 gene expression levels of the nine oxidative stress genes. Percent predicted lung function was defined as the ratio of observed lung function over predicted lung function and predicted lung function was calculated using the Hankinson equation for the corresponding age, sex, race, and height of the participants (Hankinson et al., 1999). Multivariable linear regression models were used to assess the association of lung function at CARDIA exam year 30 and 10‐year decline in lung function with year 25 gene expression levels after adjustment for center, cigarette pack‐years, and BMI. Sensitivity analysis was performed by removing participants with asthma and COPD at years 20 and 30, and evaluation of the association of lung function at CARDIA exam year 30 and 10‐year decline in lung function with year 25 gene expression levels of the nine oxidative stress genes in the subset of participants without COPD/asthma. All the *p*‐values ≤0.05 were considered statistically significant. Statistical analyses were carried out using SAS software version 9.4 (SAS Institute).

## 3 RESULTS

### 3.1 Characteristics at year 25 examination

The participants in the highest quartile of *NDUFB3* were more likely to be female (69.37% vs. 51.94%; *p*‐value <0.0001), younger (49.11 vs 50.32; *p*‐value = 0.005), current smokers (16.37% vs. 10.07%; *p*‐value = 0.03), have higher BMI (31.87 vs. 28.88; *p*‐value <0.0001), and higher C‐reactive protein (4.35 vs. 2.14; *p*‐value <0.0001). Current smokers had higher pack‐years in the fourth quartile of *NDUFB3* (21.21 vs. 16.58; *p*‐value = 0.02). Participants in the highest quartile of *MPO* were more likely to be male, participants in the highest quartile of *ALOX12* were more likely to be male and have lower C‐reactive protein, participants in the highest quartile of *PLA2G7* were more likely to be White, have lower BMI and C‐reactive protein (data in Tables S1a‐1h, Table 1).

**TABLE 1:** Participant characteristics at year 25 with respect to *NDUFB3* gene expression levels

Characteristics	*NDUFB3* gene expression levels				
	0–25 percentile (n = 566)	>25 to 50 percentile (n = 570)	>50 to 75 percentile (n = 567)	>75 to 100 percentile (n = 568)	*p*‐value
Age (years)	50.32 (3.56)	50.29(3.48)	50.24 (3.60)	49.73(3.62)	0.02
Race					0.05
%Blacks	42.76	40.53	40.56	47.71	
Sex					<0.0001
% Female	51.94	53.16	59.26	69.37	
Smoking					0.03
% Never	63.78	67.02	64.55	63.91	
% Former	26.15	21.40	22.05	19.72	
Smoking pack‐years among former smokers	7.16 ± 7.97	7.39 ± 8.86	7.48 ± 8.16	7.74 ± 8.64	0.96
% Current	10.07	11.58	13.40	16.37	
Smoking pack‐years among current smokers	16.58 ± 11.81	16.89 ± 11.56	20.18 ± 11.48	21.21 ± 13.63	0.02
BMI	28.89 (6.12)	29.22 (6.53)	29.36 (6.77)	32.19 (8.10)	<0.0001
Alcohol consumption (ml/day)	11.95 (24.76)	10.66 (16.81)	11.06 (17.19)	9.40 (20.38)	0.40
C‐reactive protein (µG/ML)	2.14 (3.15)	2.56 (4.25)	2.78 (5.07)	4.35 (5.80)	<0.0001

### 3.2 Association between year 30 lung function and year 25 gene expression profiles

Year 30 predicted FVC was nominally lower in the highest quartile of *NDUFB3* as compared to the lowest level of *NDUFB3*, with a difference of 2.30% (95% CI: 06.8%‐3.93%; *p*‐value = 0.04; Table 2). None of the other genes were associated with year 30 FEV_1_ or FVC (Table S2). A *p*‐value of 0.003 (nine markers with two outcomes = 0.05/18 = 0.003) to determine statistical significance using Bonferroni correction for multiple comparisons indicates that the associations are not statistically significant.

**TABLE 2:** Association between year 30 lung function and year 25 gene expression levels

Markers	First quartile	Second quartile	Third quartile	Fourth quartile	Difference between first and final levels	*p*‐value for trend
Year 30%predicted FEV_1_						
*ARG2*	92.90 ± 0.64	92.34 ± 0.64	92.41 ± 0.64	92.46 ± 0.64	0.44 (−1.21, 2.33)	0.54
*NDUFB3*	92.94 ± 0.64	92.87 ± 0.64	93.01 ± 0.64	91.31 ± 0.64	1.63 (−0.17, 3.42)	0.08
*PLA2G7*	91.49 ± 0.64	92.22 ± 0.64	93.26 ± 0.64	93.16 ± 0.64	−1.68 (−3.46, 0.10)	0.08
Year 30%predicted FVC						
*ARG2*	94.88 ± 0.58	94.14 ± 0.58	94.26 ± 0.58	93.74 ± 0.58	1.14 (−0.47, 2.76)	0.29
*NDUFB3*	95.05 ± 0.58	94.53 ± 0.58	94.69 ± 0.58	92.75 ± 0.58	2.30 (0.68, 3.93)	0.04
*PLA2G7*	93.36 ± 0.58	94.12 ± 0.58	95.15 ± 0.58	94.40 ± 0.58	−1.04 (−2.66, 0.58)	0.25

Note

All percent predicted estimates are represented as percentage ± SE. The differences are represented with the 95% CI. Linear regression models with adjustment for center, smoking pack‐years, and BMI.

### 3.3 Association between 10‐year decline in lung function and year 25 gene expression profiles

Decline in FEV_1_ from year 20 to year 30 was higher in the highest quartile of *NDUFB3* as compared to the lowest quartile of *NDUFB3* (3.73% vs. 1.64%; *p*‐value = 0.001). Decline in FVC from year 20 to year 30 was nominally higher in the highest quartile of *ARG2* as compared to the lowest level of *ARG2* (3.79% vs. 2.48%; *p*‐value = 0.02). Decline in FEV_1_ and FVC was nominally lower in the highest quartile of *PLA2G7* as compared to the lowest quartile (2.21% vs. 3.35%; *p*‐value = 0.02 for FEV_1_ and 2.62% vs. 3.86%; *p*‐value = 0.005; Table 3). None of the other genes were associated with 10‐year decline in lung function from year 20 to year 30 (Table S3). After adjustment for multiple comparisons using Bonferroni correction (a corrected *p*‐value of 0.003) only the association between *NDUFB3* and 10‐year decline in FEV_1_ remained statistically significant.

**TABLE 3:** Association between 10‐year change in lung function from year 20 to year 30 and year 25 gene expression profiles

Markers	First quartile	Second quartile	Third quartile	Fourth quartile	Difference between first and final levels	*p*‐value for trend
% predicted FEV1—10‐year decline						
*ARG2*	2.41 ± 0.40	1.78 ± 0.40	2.61 ± 0.40	3.29 ± 0.40	−0.87 (−1.99, 0.25)	0.08
*NDUFB3*	1.64 ± 0.49	2.39 ± 0.40	2.37 ± 0.40	3.73 ± 0.41	−2.09 (−3.22, −0.95)	0.001
*PLA2G7*	3.35 ± 0.41	2.37 ± 0.40	2.20 ± 0.40	2.21 ± 0.40	1.15 (0.02, 2.27)	0.02
% predicted FVC—10‐year decline						
*ARG2*	2.48 ± 0.40	2.50 ± 0.40	2.84 ± 0.40	3.79 ± 0.40	−1.31 (−2.42, −0.20)	0.02
*NDUFB3*	2.35 ± 0.40	2.81 ± 0.39	2.84 ± 0.40	3.65 ± 0.41	−1.30 (−2.43, −0.18)	0.07
*PLA2G7*	3.86 ± 0.40	2.69 ± 0.40	2.48 ± 0.40	2.62 ± 0.39	1.24 (0.12, 2.35)	0.005

Note

All percent predicted estimates are represented as percentage ± SE. The differences are represented with the 95% CI. Linear regression models with adjustment for center, smoking pack‐years, and BMI.

### 3.4 Sensitivity analysis after exclusion of asthma and COPD patients

All *ARG2* and *NDUFB3* quartiles had a similar distribution of asthma and COPD patients (ARG2: 19.37% vs. 19.18%; *p* = 0.64 and 1.90% vs. 2.54%; *p* = 0.81 and *NDUFB3*: 18.1% vs. 21.11%; *p* = 0.08 and 1.59% vs. 2.70%; *p* = 0.58). The distribution of asthma across *PLA2G7* quartiles was different (22.19% vs. 14.13%; *p* = 0.001) and distribution of COPD across quartiles of *PLA2G7* was similar (2.85% vs. 1.27%; *p* = 0.27). Eliminating asthma and COPD patients from the analysis did not substantially change the observed associations. Year 30 FVC was lower in the fourth *NDUFB3* quartile versus the first *NDUFB3* quartile (difference: 2.69% [95% CI: 0.94, 4.45]; *p* = 0.01). The 10‐year decline of FEV_1_ was higher in the highest *NDUFB3* level versus the first *NDUFB3* quartile (difference: −1.69% (95% CI: −2.89, −0.50); *p* = 0.02) and the 10‐year decline of FVC was lower in the highest *PLA2G7* level versus the first *PLA2G7* quartile (difference: 1.03% [95% CI: −0.14, 2.21]; *p* = 0.01).

## 4 DISCUSSION

This study found that faster 10‐year decline in FEV_1_ was associated with higher *NDUFB3* gene expression levels after adjusting for multiple comparisons using Bonferroni correction. Faster 10‐year decline in FVC was nominally associated with higher expression of *ARG2* and faster 10‐year decline in FEV_1_ and FVC were nominally associated with lower *PLA2G7* though both these associations were no longer significant after adjustment for multiple comparisons. For most part, the results for *NDUFB3*, *ARG2*, and *PLA2G7* are consistent with our hypothesis that higher gene expression levels are associated with lower lung function. The other six genes, which were included in these analyses were not associated with FEV_1_ and FVC.

Previous studies on oxidative stress and lung function have measured markers such as *p*‐TBARS in LDL cholesterol and Glutathione (GSH) in blood and plasma to study associations with FEV_1_ and FVC. A study done in 137 nonsmokers found that *p*‐TBARS was negatively associated with %FEV_1_ (*p*‐value = 0.02), indicating the role of lipid peroxidation in lung health (Schunemann et al., 1997). Another study also reported an inverse association between TBARS and %FVC (*p*‐value = 0.02; Ochs‐Balcom et al., 2005). In addition, dietary antioxidants such as Vitamin C, Vitamin E, and Lutein/zeaxanthin were positively associated with %FEV_1_ and %FVC (Ochs‐Balcom et al., 2005). However, gene expression levels of enzymes that affect oxidative stress have not been evaluated previously.

Our findings suggest that higher levels of expression of *NDUFB3* was associated with 10‐year decline in FEV_1_ and nominally associated with lower year 30 percent predicted FVC. *NDUFB3* is one of the genes involved in the oxidoreductase genes involved in the NADH dehydrogenase: ubiquinone complex I, which is a mitochondrial subunit needed for electron transfer. Consistent with our findings, a previous study has found an upregulation in these cluster of oxidoreductase genes, involved in complex I, among individuals with severe cystic fibrosis (CF) lung disease compared with mild CF disease and non‐CF control subjects (Wright et al., 2006). Upregulated levels of arginase have been found to be associated with pulmonary diseases like asthma, COPD, and cystic fibrosis (Bratt et al., 2011 Sep; Maarsingh et al., 2008,). Although cystic fibrosis, asthma, and COPD have different pathophysiology, lower lung function, and accelerated decline in lung function has been observed in these three diseases (James et al., 2005; Peat et al., 1987; Tantucci & Modina, 2012; Vandenbranden et al., 2012). In a childhood asthma study done among 433 case‐parent triads, genetic variation in *ARG2* had an increased risk of childhood asthma (Li et al., 2006). Consistent with these findings, we found that faster 10‐year decline of percent predicted FVC was associated with higher level of *ARG2*. Previous studies have found that deficiency of *PLA2G7*, which occurred due to a missense mutation that resulted in complete loss of activity, was found to be higher among asthmatics in a Japanese population (Stafforini et al., 1999). Two other variants in PAF‐AH were also associated with asthma in Caucasian population, and deficiency in serum PAF‐AH was higher among asthmatic children (Kruse et al., 2000; Miwa et al., 1988). Consistent with these findings, we found that 10‐year decline of FEV_1_ and FVC was slower in the highest levels of *PLA2G7*. However, increased expression of PAF‐AH is also associated with release of components such as free F2‐isoprostanes which increase oxidative stress. We hypothesize that the action of PAF‐AH is dependent on the local environment and the specific biological effect of PLA2G7 on lung health will need to be clarified in future studies.

Long‐term follow‐up of participants and representative sample with inclusion of men and women, and Black and White participants are some of the strengths of the study. Gene expression measurements of biomarkers are useful when protein measurements of biomarkers are not available. Our study has several limitations such as lung function measurements and gene expression measurements being performed in different years, restricting our understanding of the temporal relationship between gene expression of biomarkers and lung function and gene expression measurements are available at a single time point, limiting our ability to study the longitudinal relationship with lung function. In addition, different methods used for measuring FEV_1_ and FVC at year 20 (a dry rolling‐seal OMI spirometer) and year 30 (portable spirometer) could have influenced the measurements. However, measurements at both time points were performed following the ATS guidelines reducing the variation across both measurements. Gene expression levels of these oxidative stress markers could be correlated with differences in cell composition such as the proportion of monocytes, T‐lymphocytes. Since complete blood counts are not available in CARDIA at year 25, differences in cell composition may be a potential confounder in the observed association. The observed results indicate an association between higher gene expression levels of NDUFB3 and faster decline in FEV1 and possible associations between *ARG2*, *PLA2G7*, and lung function. These findings need to be confirmed in independent studies.

In conclusion, these results suggest that high levels of gene expression of these markers are associated with lower lung function, independent of cigarette smoking, and BMI. Hence, measuring gene expression levels of other markers in mitochondrial dysfunction pathways and arginine pathways at multiple time points in independent datasets may help us identify the genes involved in lung function decline and understand how these pathways affect lung health.

## CONFLICT OF INTEREST

The authors declare that they have no conflict of interest.

## AUTHOR CONTRIBUTIONS

R.R. worked on the data analysis and drafted the manuscript; R.K., D.J., L.H., G.W., and B.T. helped with the critical review of the analysis and manuscript; M.G. conducted the gene expression measurement experiments; W.G. conducted the data analysis for the gene expression measurements.

## Supporting information

Table S1‐S3

## Data Availability

Research data are not shared.
