# Supplementary material for: Gene expression of oxidative stress markers and lung function: A CARDIA lung study
Source: Mol Genet Genomic Med. 2021 Nov 19;9(12):e1832. doi: 10.1002/mgg3.1832 (PMC8683624; doi:10.1002/mgg3.1832)
Supplement: Supplementary file 1 — Table S1‐S3 [file MGG3-9-e1832-s001.docx]

GENE EXPRESSION OF OXIDATIVE STRESS MARKERS AND LUNG FUNCTION: A CARDIA LUNG STUDY

Ramya Ramasubramanian^1^, Ravi Kalhan^2,3^, David R. Jacobs Jr.^1^, George R. Washko^4,5^, Lifang Hou^2^, Myron D. Gross^6^, Weihua Guan^7^, Bharat Thyagarajan^6,8^.

1. Division of Epidemiology and Community Health, University of Minnesota School of Public Health, Minneapolis, MN, USA
2. Department of Preventive Medicine, Northwestern University Feinberg School of Medicine, Chicago, IL, USA
3. Division of Pulmonary and Critical Care Medicine, Northwestern University Feinberg School of Medicine, Chicago, IL, USA
4. Division of Pulmonary and Critical Care Medicine, Brigham and Women’s Hospital, Boston, MA, USA
5. Applied Chest Imaging Laboratory, Brigham and Women’s Hospital, Boston, MA, USA
6. Department of Pathology and Laboratory Medicine, University of Minnesota School of Medicine, Minneapolis, MN, USA
7. Department of Biostatistics, University of Minnesota School of Public Health, Minneapolis, MN, USA

**Supplementary table 1a: Participant characteristics at year 25 with respect to *ARG2* gene expression levels**

| **Characteristics** | *ARG2* gene expression levels | | | | **p-value** |
| --- | --- | --- | --- | --- | --- |
|  | 0-25 percentile  (n=567) | >25-50 percentile  (n=567) | >50-75 percentile  (n=568) | >75-100 percentile  (n=569) |  |
| Age (years) | 50.18 (3.56) | 50.44 (3.47) | 50.04 (3.59) | 49.93 (3.65) | 0.09 |
| Race |  |  |  |  |  |
| %Blacks | 30.51 | 35.80 | 45.60 | 59.58 | <0.0001 |
| Sex |  |  |  |  |  |
| % Female | 43.74 | 56.79 | 64.61 | 68.54 |  |
| Smoking |  |  |  |  | 0.42 |
| Never | 64.73 | 62.96 | 65.49 | 66.08 |  |
| Former | 20.81 | 24.51 | 20.95 | 23.02 |  |
| Current | 14.46 | 12.52 | 13.56 | 10.90 |  |
| BMI | 29.13 (6.42) | 29.55 (7.15) | 30.18 (7.18) | 30.81 (7.30) | 0.0003 |
| Alcohol consumption (mL/day) | 13.80 (21.40) | 12.54 (25.20) | 9.21 (15.97) | 8.10 (15.45) | <0.0001 |
| C-reactive protein (uG/ML) | 2.21 (3.52) | 3.14 (5.46) | 2.94 (4.14) | 3.55 (5.47) | <0.0001 |

**Supplementary table 1b: Participant characteristics at year 25 with respect to *PLA2G7* gene expression levels**

| **Characteristics** | *PLA2G7* gene expression levels | | | | **p-value** |
| --- | --- | --- | --- | --- | --- |
|  | 0-25 percentile  (n=567) | >25-50 percentile  (n=568) | >50-75 percentile  (n=568) | >75-100 percentile  (n=568) |  |
| Age (years) | 49.68 (3.81) | 49.90 (3.55) | 50.45 (3.56) | 50.55 (3.29) | <0.0001 |
| Race |  |  |  |  | <0.0001 |
| %Blacks | 75.49 | 47.18 | 33.45 | 15.49 |  |
| Sex |  |  |  |  | <0.0001 |
| % Female | 32.98 | 39.44 | 43.66 | 50.18 |  |
| Smoking |  |  |  |  | 0.59 |
| Never | 63.84 | 65.14 | 67.25 | 63.03 |  |
| Former | 21.87 | 22.54 | 20.25 | 24.65 |  |
| Current | 14.29 | 12.32 | 12.50 | 12.32 |  |
| BMI | 31.15 (7.23) | 30.66 (7.36) | 29.53 (6.90) | 28.33 (6.31) | <0.0001 |
| Alcohol consumption (mL/day) | 9.23 (22.89) | 8.73 (14.22) | 12.23 (22.10) | 13.43 (19.38) | <0.0001 |
| C-reactive protein (uG/ML) | 3.62 (5.21) | 3.18 (4.45) | 2.68 (4.08) | 2.37 (5.08) | <0.0001 |

**Supplementary table 1c: Participant characteristics at year 25 with respect to *ALOX12* gene expression levels**

| **Characteristics** | *ALOX12* gene expression levels | | | | **p-value** |
| --- | --- | --- | --- | --- | --- |
|  | 0-25 percentile  (n=567) | >25-50 percentile  (n=568) | >50-75 percentile  (n=569) | >75-100 percentile  (n=567) |  |
| Age (years) | 49.84 (3.59) | 50.31 (3.56) | 50.15 (3.49) | 50.28 (3.64) | <0.0001 |
| Race |  |  |  |  | 0.02 |
| %Blacks | 38.45 | 41.55 | 44.64 | 46.91 |  |
| Sex |  |  |  |  | 0.001 |
| % Female | 53.26 | 55.63 | 62.74 | 62.08 |  |
| Smoking |  |  |  |  | 0.001 |
| Never | 62.43 | 60.56 | 67.84 | 68.43 |  |
| Former | 21.87 | 23.59 | 20.74 | 23.10 |  |
| Current | 15.70 | 15.85 | 11.42 | 8.47 |  |
| BMI | 28.52 (6.28) | 29.90 (7.03) | 30.37 (7.24) | 30.86 (7.37) | <0.0001 |
| Alcohol consumption (mL/day) | 12.60 (26.19) | 11.39 (18.55) | 9.43 (15.42) | 10.22 (18.30) | 0.04 |
| C-reactive protein (uG/ML) | 2.23 (3.67) | 2.95 (4.76) | 3.29 (5.22) | 3.37 (5.10) | 0.0002 |

**Supplementary table 1d: Participant characteristics at year 25 with respect to *ALOX12* gene expression levels**

| **Characteristics** | *ALOX15* gene expression levels | | | | **p-value** |
| --- | --- | --- | --- | --- | --- |
|  | 0-25 percentile  (n=567) | >25-50 percentile  (n=568) | >50-75 percentile  (n=569) | >75-100 percentile  (n=567) |  |
| Age (years) | 49.97 (3.74) | 49.89 (3.51) | 50.35 (3.57) | 50.37 (3.45) | 0.04 |
| Race |  |  |  |  | <0.0001 |
| %Blacks | 53.09 | 45.07 | 39.72 | 33.69 |  |
| Sex |  |  |  |  | 0.005 |
| % Female | 63.32 | 59.15 | 58.35 | 52.91 |  |
| Smoking |  |  |  |  | 0.42 |
| Never | 67.90 | 66.37 | 63.27 | 61.73 |  |
| Former | 20.63 | 21.13 | 23.37 | 24.16 |  |
| Current | 11.46 | 12.50 | 13.36 | 14.11 |  |
| BMI | 29.95 (6.79) | 30.75 (7.55) | 29.90 (7.11) | 29.06 (6.61) | 0.001 |
| Alcohol consumption (mL/day) | 10.98 (20.26) | 9.81 (17.13) | 10.45 (22.25) | 12.39 (20.13) | 0.16 |
| C-reactive protein (uG/ML) | 3.30 (5.76) | 3.25 (4.96) | 2.70 (3.88) | 2.59 (4.12) | 0.02 |

**Supplementary table 1e: Participant characteristics at year 25 with respect to *GSTT1* gene expression levels**

| **Characteristics** | *GSTT1* gene expression levels | | | | **p-value** |
| --- | --- | --- | --- | --- | --- |
|  | 0-25 percentile  (n=567) | >25-50 percentile  (n=568) | >50-75 percentile  (n=569) | >75-100 percentile  (n=567) |  |
| Age (years) | 50.04 (3.53) | 50.06 (3.62) | 50.19 (3.57) | 50.29 (3.58) | 0.59 |
| Race |  |  |  |  | <0.0001 |
| %Blacks | 48.85 | 34.86 | 39.02 | 48.85 |  |
| Sex |  |  |  |  | 0.80 |
| % Female | 58.73 | 57.22 | 57.82 | 59.96 |  |
| Smoking |  |  |  |  | 0.16 |
| Never | 64.55 | 63.73 | 67.31 | 63.67 |  |
| Former | 20.11 | 23.77 | 22.67 | 22.75 |  |
| Current | 15.34 | 12.50 | 10.02 | 13.58 |  |
| BMI | 30.33 (7.10) | 29.96 (7.24) | 29.52 (6.89) | 29.86 (6.94) | 0.28 |
| Alcohol consumption (mL/day) | 11.18 (20.79) | 10.03 (15.44) | 11.56 (24.88) | 10.85 (17.77) | 0.62 |
| C-reactive protein (uG/ML) | 3.43 (5.37) | 2.81 (4.50) | 2.90 (4.85) | 2.70 (4.16) | 0.05 |

**Supplementary table 1f: Participant characteristics at year 25 with respect to *SOD3* gene expression levels**

| **Characteristics** | *SOD3* gene expression levels | | | | **p-value** |
| --- | --- | --- | --- | --- | --- |
|  | 0-25 percentile  (n=564) | >25-50 percentile  (n=571) | >50-75 percentile  (n=569) | >75-100 percentile  (n=567) |  |
| Age (years) | 50.10 (3.58) | 50.21 (3.56) | 49.91 (3.66) | 50.36 (3.48) | 0.19 |
| Race |  |  |  |  | <0.0001 |
| %Blacks | 35.64 | 36.43 | 48.33 | 51.15 |  |
| Sex |  |  |  |  | 0.002 |
| % Female | 52.48 | 57.97 | 59.58 | 63.67 |  |
| Smoking |  |  |  |  | 0.38 |
| Never | 63.12 | 63.05 | 64.85 | 68.25 |  |
| Former | 23.23 | 22.94 | 23.73 | 19.40 |  |
| Current | 13.65 | 14.01 | 11.42 | 12.35 |  |
| BMI | 29.44 (6.72) | 29.89 (7.02) | 30.24 (7.39) | 30.07 (7.02) | 0.25 |
| Alcohol consumption (mL/day) | 12.60 (20.72) | 11.62 (24.12) | 10.52 (18.42) | 8.89 (15.75) | 0.01 |
| C-reactive protein (uG/ML) | 2.74 (4.45) | 3.01 (5.31) | 2.99 (4.46) | 3.10 (4.72) | 0.61 |

**Supplementary table 1g: Participant characteristics at year 25 with respect to *LPO* gene expression levels**

| **Characteristics** | *LPO* gene expression levels | | | | **p-value** |
| --- | --- | --- | --- | --- | --- |
|  | 0-25 percentile  (n=574) | >25-50 percentile  (n=568) | >50-75 percentile  (n=559) | >75-100 percentile  (n=570) |  |
| Age (years) | 50.13 (3.57) | 50.35 (3.50) | 49.86 (3.65) | 50.24 (3.57) | 0.13 |
| Race |  |  |  |  | <0.0001 |
| %Blacks | 34.32 | 37.32 | 50.45 | 49.65 |  |
| Sex |  |  |  |  | 0.51 |
| % Female | 56.79 | 57.04 | 59.39 | 60.53 |  |
| Smoking |  |  |  |  | 0.37 |
| Never | 62.89 | 64.61 | 63.69 | 68.07 |  |
| Former | 23.00 | 24.12 | 22.36 | 19.82 |  |
| Current | 14.11 | 11.27 | 13.95 | 12.11 |  |
| BMI | 29.74 (6.96) | 29.34 (6.74) | 29.97 (6.93) | 30.60 (7.48) | 0.03 |
| Alcohol consumption (mL/day) | 12.34 (21.08) | 11.34 (17.57) | 9.97 (18.06) | 9.95 (22.84) | 0.13 |
| C-reactive protein (uG/ML) | 2.99 (4.75) | 2.75 (4.45) | 3.04 (5.13) | 3.07 (4.65) | 0.67 |

**Supplementary table 1h: Participant characteristics at year 25 with respect to *MPO* gene expression levels**

| **Characteristics** | *MPO* gene expression levels | | | | **p-value** |
| --- | --- | --- | --- | --- | --- |
|  | 0-25 percentile  (n=569) | >25-50 percentile  (n=567) | >50-75 percentile  (n=568) | >75-100 percentile  (n=567) |  |
| Age (years) | 50.12 (3.70) | 50.19 (3.55) | 50.15 (3.52) | 50.13 (3.52) | 0.99 |
| Race |  |  |  |  | 0.98 |
| %Blacks | 43.23 | 42.86 | 43.31 | 42.15 |  |
| Sex |  |  |  |  | <0.0001 |
| % Female | 62.04 | 60.67 | 61.44 | 49.56 |  |
| Smoking |  |  |  |  | 0.99 |
| Never | 63.97 | 65.61 | 65.49 | 64.20 |  |
| Former | 23.20 | 21.69 | 21.30 | 23.10 |  |
| Current | 12.83 | 12.70 | 13.20 | 12.70 |  |
| BMI | 30.01 (6.94) | 29.67 (7.13) | 29.64 (7.06) | 30.34 (7.04) | 0.29 |
| Alcohol consumption (mL/day) | 10.64 (24.39) | 9.98 (17.74) | 11.33 (19.38) | 11.68 (17.89) | 0.49 |
| C-reactive protein (uG/ML) | 2.80 (4.16) | 2.93 (5.05) | 3.11 (4.83) | 2.99 (4.90) | 0.74 |

| Year 30 % predicted FEV1 | | | | | | |
| --- | --- | --- | --- | --- | --- | --- |
| Markers | First quartile | Second quartile | Third quartile | Fourth quartile | Difference between first and final quartiles | p-value for trend |
| *ALOX12* | 92.53 ± 0.63 | 91.75 ± 0.03 | 92.99 ± 0.62 | 92.48 ± 0.63 | 0.05 (-1.70, 1.80) | 0.86 |
| *ALOX15* | 91.99 ± 0.62 | 92.70 ± 0.62 | 93.33 ± 0.62 | 91.72 ± 0.63 | 0.27 (-1.47, 2.00) | 0.85 |
| *LPO* | 92.46 ± 0.62 | 91.93 ± 0.63 | 92.57 ± 0.63 | 92.78 ± 0.62 | -0.32 (-2.05, 1.41) | 0.66 |
| *MPO* | 92.26 ± 0.63 | 92.22 ± 0.62 | 93.46 ± 0.63 | 91.81 ± 0.62 | 0.45 (-1.29, 2.18) | 0.79 |
| *GSTT1* | 92.58 ± 0.63 | 91.92 ± 0.62 | 92.18 ± 0.62 | 93.06 ± 0.62 | -0.49 (-2.22, 1.25) | 0.67 |
| *SOD3* | 91.66 ± 0.62 | 91.99 ± 0.63 | 93.44 ± 0.63 | 92.68 ± 0.62 | -1.02 (-2.74, 0.71) | 0.21 |
| Year 30 % predicted FVC | | | | | | |
| *ALOX12* | 94.38 ± 0.57 | 93.90 ± 0.57 | 94.60 ± 0.56 | 93.88 ± 0.57 | 0.50 (-1.08, 2.08) | 0.62 |
| *ALOX15* | 93.65 ± 0.57 | 94.24 ± 0.57 | 94.79 ± 0.57 | 94.08 ± 0.57 | -0.43 (-1.99, 1.14) | 0.35 |
| *LPO* | 94.37 ± 0.56 | 93.72 ± 0.57 | 94.23 ± 0.57 | 94.42 ± 0.57 | -0.05 (-1.62, 1.51) | 0.94 |
| *MPO* | 94.30 ± 0.57 | 94.32 ± 0.56 | 94.80 ± 0.57 | 93.34 ± 0.56 | 0.96 (-0.60, 2.53) | 0.34 |
| *GSTT1* | 93.73 ± 0.57 | 94.22 ± 0.56 | 93.79 ± 0.56 | 95.02 ± 0.56 | -1.29 (-2.86, 0.27) | 0.20 |
| *SOD3* | 93.80 ± 0.56 | 93.83 ± 0.57 | 95.06 ± 0.57 | 94.08 ± 0.56 | -0.28 (-1.84, 1.28) | 0.74 |
| Year 30 % predicted FEV1/ % predicted FVC | | | | | | |
| *ALOX12* | 98.09 ± 0.34 | 97.74 ± 0.34 | 98.41 ± 0.34 | 98.49 ± 0.34 | -0.40 (-1.34,0.54) | 0.30 |
| *ALOX15* | 98.28 ± 0.34 | 98.49 ± 0.34 | 98.55 ± 0.34 | 97.42 ± 0.34 | 0.86 (-0.07, 1.79) | 0.16 |
| *LPO* | 98.12 ± 0.33 | 98.09 ± 0.34 | 98.25 ± 0.34 | 98.28 ± 0.34 | -0.16 (-1.09, 0.77) | 0.40 |
| *MPO* | 97.85 ± 0.34 | 97.92 ± 0.33 | 98.63 ± 0.34 | 98.34 ± 0.34 | -0.49 (-1.42, 0.44) | 0.32 |
| *GSTT1* | 98.85 ± 0.34 | 97.64 ± 0.34 | 98.31 ± 0.34 | 97.94 ± 0.34 | 0.91 (-0.02, 1.84) | 0.11 |
| *SOD3* | 97.83 ± 0.33 | 97.88 ± 0.34 | 98.39 ± 0.35 | 98.63 ± 0.34 | -0.80 (-1.73, 0.12) | 0.04 |

**Supplementary table 2: Association between year 30 lung function and year 25 gene expression levels**

**Supplementary table 3: Association between 10-year change in lung function from year 20 to year 30 and year 25 gene expression profiles**

| % predicted FEV1 – 10-year decline | | | | | | |
| --- | --- | --- | --- | --- | --- | --- |
| Markers | First quartile | Second quartile | Third quartile | Fourth quartile | Difference between first and final quartiles | p-value for trend |
| *ALOX12* | 2.83 ± 0.39 | 2.52 ± 0.39 | 2.32 ± 0.39 | 2.51 ± 0.39 | 0.32 (-0.77, 1.42) | 0.64 |
| *ALOX15* | 2.83 ± 0.39 | 2.35 ± 0.39 | 1.92 ± 0.39 | 3.08 ± 0.39 | -0.25 (-1.34, 0.83) | 0.68 |
| *LPO* | 2.71 ± 0.39 | 2.42 ± 0.39 | 2.71 ± 0.39 | 2.35 ± 0.39 | 0.35 (-0.73, 1.44) | 0.89 |
| *MPO* | 2.89 ± 0.39 | 2.34 ± 0.39 | 2.12 ± 0.39 | 2.83 ± 0.39 | 0.05 (-1.03, 1.14) | 0.48 |
| *GSTT1* | 2.22 ± 0.39 | 2.95 ± 0.39 | 2.40 ± 0.39 | 2.61 ± 0.39 | -0.38 (-1.47, 0.70) | 0.59 |
| *SOD3* | 2.78 ± 0.39 | 2.49 ± 0.39 | 2.82 ± 0.40 | 2.86 ±  0.39 | -0.07 (-1.15, 1.00) | 0.98 |
| % predicted FVC – 10-year decline | | | | | | |
| *ALOX12* | 3.08 ± 0.39 | 2.73 ± 0.39 | 2.54 ± 0.39 | 3.30 ± 0.39 | -0.22 (-1.31, 0.86) | 0.66 |
| *ALOX15* | 3.28 ± 0.39 | 2.57 ± 0.39 | 2.46 ± 0.39 | 3.34 ± 0.39 | -0.07 (-1.14, 1.01) | 0.96 |
| *LPO* | 2.85 ± 0.38 | 2.81 ± 0.39 | 2.87 ± 0.39 | 3.11 ± 0.39 | -0.27 (-1.34, 0.81) | 0.66 |
| *MPO* | 3.44 ± 0.39 | 2.65 ± 0.39 | 2.37 ± 0.39 | 3.17 ± 0.39 | 0.26 (-0.82, 1.33) | 0.30 |
| *GSTT1* | 2.69 ± 0.39 | 2.79 ± 0.39 | 3.14 ± 0.39 | 3.03 ± 0.39 | -0.34 (-1.41, 0.73) | 0.65 |
| *SOD3* | 3.02 ± 0.39 | 3.07 ± 0.38 | 3.04 ± 0.39 | 3.03 ± 0.38 | -0.01 (-1.08, 1.06) | 0.86 |
| % predicted FEV1/ % predicted FVC – 10-year decline | | | | | | |
| *ALOX12* | -0.77 ± 0.23 | -0.79 ± 0.22 | -0.94 ± 0.22 | -1.29 ± 0.23 | 0.52 (-0.11, 1.14) | 0.07 |
| *ALOX15* | -1.14 ± 0.22 | -0.83 ± 0.22 | -1.14 ± 0.22 | -0.68 ± 0.22 | -0.46 (-1.08, 0.16) | 0.13 |
| *LPO* | -0.80 ± 0.22 | -0.90 ± 0.22 | -0.80 ± 0.23 | -1.28 ± 0.22 | 0.48 (-0.14, 1.10) | 0.37 |
| *MPO* | -0.99 ± 0.22 | -1.06 ± 0.22 | -0.95 ± 0.22 | -0.79 ± 0.22 | -0.20 (-0.82, 0.43) | 0.45 |
| *GSTT1* | -1.11 ± 0.22 | -0.39 ± 0.22 | -1.26 ± 0.22 | -1.04 ± 0.22 | -0.08 (-0.70, 0.54) | 0.82 |
| *SOD3* | -0.84 ± 0.22 | -0.87 ± 0.22 | -0.89 ± 0.23 | -0.85 ± 0.22 | 0.02 (-0.60, -0.64) | 0.65 |
